# Editorial: The Role of Sex Dimorphism in Disease Susceptibility and Immune Response

**DOI:** 10.3389/fnut.2022.849563

**Published:** 2022-02-07

**Authors:** Hongji Dai, Xi Zhang, Wen Zhang, Zheng Wang, Miaozhen Qiu

**Affiliations:** ^1^Key Laboratory of Molecular Cancer Epidemiology of Tianjin, Department of Epidemiology and Biostatistics, National Clinical Research Center for Cancer, Tianjin Medical University Cancer Institute and Hospital, Tianjin, China; ^2^Clinical Research Unit, Xinhua Hospital Affiliated to Shanghai Jiao Tong University School of Medicine, Shanghai, China; ^3^Department of Social Preventive Medical Sciences, Center for Preventive Medical Sciences, Chiba University, Chiba, Japan; ^4^Department of Epidemiology and Population Health, Albert Einstein College of Medicine, Bronx, NY, United States; ^5^Department of Medical Oncology, Sun Yat-sen University Cancer Center, Guangzhou, China; ^6^State Key Laboratory of Oncology in South China, Guangzhou, China; ^7^Collaborative Innovation Center for Cancer Medicine, Guangzhou, China

**Keywords:** sex disparity, hormone, cancer epidemiology, prevention, survival

Sexual dimorphism (SDM) refers to a series of differences between males and females in the same species. In humans, besides significant differences in morphology, body mass, and nutrient metabolism between men and women, persistent evidence suggests that sex disparities exist in the most non-communicable diseases as well as certain infectious diseases. For instance, the age-standardized cancer incidence rates at shared sites are substantially higher in men than in women (1). Also, women less often received systemic treatment compared with men for some cancers (2). The SDM is also observed in cardiovascular disease (CVD) in relation to incidence and prognosis (3). Recently, a male bias in COVID-19 mortality in nearly all countries with available sex-disaggregated data is observed, and the risk of death in males is ~1.7 times higher than in females (4). Sex differences are intertwined with differences in gender roles socially, with behavioral factors, and pathophysiology, which can also influence diseases develop and progress. However, the detailed underlying mechanisms for these sex differences in health and many diseases remain poorly understood.

Genetic polymorphisms could be one of the primary underlying mechanisms explaining gender difference in disease susceptibility and immune response. Li et al. investigated the potential association of leptin gene, leptin receptor gene, and peroxisome proliferator activated receptor gamma gene polymorphisms with humoral immune response to influenza vaccine. By genotype and haplotype analyses, they found that LEPR rs6673591 GA +AA genotype was correlated with low responsiveness to influenza vaccine only in males, while PPARG rs17793951 AG + GG genotype was associated with low responsiveness to influenza vaccine in females. Liu et al. used two-sample Mendelian randomization analyses to assess the causal influence of lipid-lowering agents and circulating lipid traits on sex-specific kidney cancer risk. Overall, no evidence supports the genetically proxied inhibition of HMGCR as a causal factor for renal cell carcinoma. Intriguingly, they found an association of the genetically proxied inhibition of PCSK9 and CETP with kidney cancer risk in a sex-specific manner, which should be explored further.

The first comprehensive sex-specific somatic alteration analysis of 13 cancer types from The Cancer Genome Atlas (TCGA) revealed extensive sex differences in autosomal gene expression and methylation signatures (5). Notably, 53% of clinically actionable genes show sex-biased signatures. Therefore, it is reasonable and necessary to develop sex-specific therapeutic strategies in certain cancer types. Ferroptosis has been recognized to be related to tumor growth, immune status and may have a role in regulating anti-tumor immunity (6). By pooling 6 independent cohorts with a total of 1,404 gastric cancer patients, Ma et al. revealed markedly differences of sex hormone receptors and immune cell infiltration in ferroptosis subtypes. Their newly developed ferroptosis-related score might predict the prognosis and response to immunotherapy of gastric cancer patients, especially in a subgroup of male patients.

Sex-related differences in CVD susceptibility have been well-documented in adults. A recent study indicated that CVD risk was associated with elevations from lower systolic blood pressure ranges in women compared with men (7). However, data was limited regarding the associations between early life growth patterns (e.g., birthweight) and cardiovascular outcomes. Wang et al. examined the associations between birthweight and cardiovascular parameters in children from the Shanghai Birth Cohort. They carefully assessed blood pressure, echocardiography, and anthropometry parameters, and found that macrosomia was associated with worse cardiovascular conditions in pre-school-aged boys but not in girls. Specifically, macrosomia was significantly associated with thickened left ventricle posterior wall thickness in systole and diastole, and thickened interventricular septal thickness in diastole. Boys with macrosomia showed a higher left ventricle (LV) mass index, thickened LV posterior wall thicknesses in diastole and systole and thickened interventricular septum thickness in diastole. However, no significant association with structural changes was found in girls.

It has been hypothesized that boys and girls show variations in brain development and some neurodevelopmental conditions. Fan et al. examined thyroid hormones from 452 singleton term-born infants and measured their neurodevelopment indices at the 24 months of age. Compared with the girls, the boys had a higher proportion of delay and suspected delay in the communication and personal-social domains. They also found that several alterations in thyroid hormones (e.g., free thyroxine and free triiodothyronine) of newborn boys were associated with adversely neurodevelopment, which could partly explain variations in neurodevelopment between boys and girls.

In summary, although many studies suggest both genetic and environmental factors play important and sex-specific roles in disease development, the role of gender is still obscure and in urgent need for comprehensive investigation. Currently, important knowledge gaps remain in SDM research. Considering many confounders may bias the association in epidemiological researches in relation to sex, more researches into possible biological mechanisms of male sex bias that affect the disease, particularly with respect to immune responses are warrant. Clarifying the role of gender and its molecular basis in disease susceptibility and etiology can help improve health and well-being.

## Author Contributions

HD and XZ: wrote the manuscript. All authors contributed to the article and approved the submitted version.

## Funding

HD was supported by the Tianjin Municipal Education Commission (2016YD21).

## Conflict of Interest

The authors declare that the research was conducted in the absence of any commercial or financial relationships that could be construed as a potential conflict of interest.

## Publisher's Note

All claims expressed in this article are solely those of the authors and do not necessarily represent those of their affiliated organizations, or those of the publisher, the editors and the reviewers. Any product that may be evaluated in this article, or claim that may be made by its manufacturer, is not guaranteed or endorsed by the publisher.
